# Detection of hydrogen peroxide-producing *Lactobacillus* species in the vagina: a comparison of culture and quantitative PCR among HIV-1 seropositive women

**DOI:** 10.1186/1471-2334-12-188

**Published:** 2012-08-13

**Authors:** Jennifer E Balkus, Caroline Mitchell, Kathy Agnew, Congzhou Liu, Tina Fiedler, Susan E Cohn, Amneris Luque, Robert Coombs, David N Fredricks, Jane Hitti

**Affiliations:** 1 Department of Epidemiology, University of Washington, Box 359909, 325 9th Avenue, Seattle, WA, 98104, USA; 2 Department of Obstetrics and Gynecology, University of Washington, Seattle, USA; 3 Vaccine Infectious Diseases Division, Fred Hutchinson Cancer Research Center, Seattle, USA; 4 Division of Infectious Diseases, Department of Medicine, Northwestern University, Chicago, USA; 5 Department of Medicine, University of Rochester, Rochester, USA; 6 Departments of Laboratory Medicine and Medicine, University of Washington, Seattle, USA

**Keywords:** *Lactobacillus*, *Lactobacillus crispatus*, *Lactobacillus jensenii*, Bacterial vaginosis, Culture, Hydrogen peroxide, 16S rRNA gene

## Abstract

**Background:**

The presence of hydrogen peroxide (H_2_O_2_) producing *Lactobacillus* in the vagina may play a role in controlling genital HIV-1 shedding. Sensitive molecular methods improve our ability to characterize the vaginal microbiota; however, they cannot characterize phenotype. We assessed the concordance of H_2_O_2_-producing *Lactobacillus* detected by culture with quantitative PCR (qPCR) detection of *Lactobacillus* species commonly assumed to be H_2_O_2_-producers.

**Methods:**

Samples were collected as part of a prospective cohort study of HIV-1 seropositive US women. Cervicovaginal lavage specimens were tested for *L. crispatus* and *L. jensenii* using 16S rRNA gene qPCR assays. Vaginal swabs were cultured for *Lactobacillus* and tested for H_2_O_2_-production. We calculated a kappa statistic to assess concordance between culture and qPCR.

**Results:**

Culture and qPCR results were available for 376 visits from 57 women. Lactobacilli were detected by culture at 308 (82%) visits, of which 233 of 308 (76%) produced H_2_O_2_. *L. crispatus* and/or *L. jensenii* were detected at 215 (57%) visits. Concordance between detection of *L. crispatus* and/or *L. jensenii* by qPCR and H_2_O_2_-producing *Lactobacillus* by culture was 75% (kappa = 0.45).

**Conclusions:**

Among HIV-1 seropositive women, there was a moderate level of concordance between H_2_O_2_-producing *Lactobacillus* detected by culture and the presence of *L. crispatus* and/or *L. jensenii* by qPCR. However, one-quarter of samples with growth of H_2_O_2_-producing lactobacilli did not have *L. crispatus* or *L. jensenii* detected by qPCR. This discordance may be due to the presence of other H_2_O_2_-producing *Lactobacillus* species.

## Background

Bacterial vaginosis (BV) and BV-associated organisms have been associated with an increased risk of HIV-1 RNA genital tract shedding [1,2]. Conversely, the presence of hydrogen peroxide (H_2_O_2_) producing lactobacilli in the vagina has been associated with a reduced likelihood of HIV-1 RNA shedding [2,3]. Thus, it is hypothesized that H_2_O_2_-producing lactobacilli may play an important role in controlling genital HIV-1 shedding. The development of sensitive molecular methods, such as 16S rRNA gene polymerase chain reaction (PCR) assays, improves our ability to characterize the vaginal microbiota; however, they cannot characterize H_2_O_2_ production [4]. As cultivation-independent methods become more widely used, it is important to understand how these novel methods compare to traditional culture-based methods. *Lactobacillus crispatus* and *L. jensenii* are two of the most commonly detected species of vaginal lactobacilli and the majority of strains have been found to produce H_2_O_2_[5,6]. We sought to assess the concordance of H_2_O_2_-producing *Lactobacillus* detected by culture with presumptive H_2_O_2_-producing *Lactobacillus* species detected by quantitative PCR (qPCR) among a cohort of HIV-seropositive women.

## Methods

We used samples collected as part of a prospective cohort study of HIV-1 seropositive women conducted from 2002–2007 in Seattle, WA and Rochester, NY. The institutional review boards at the University of Washington and University of Rochester approved the study and participants provided written informed consent. At enrollment, eligible women were 18–50 years old, HIV-1 seropositive and not pregnant. Women were not eligible to enroll if they had active substance abuse that would preclude their ability to participate in the study or if they had a hysterectomy. Participants had 4 study visits in the first year and 3 visits per year in subsequent years. Each study visit included a face-to-face interview to ascertain information on demographics, sexual behavior, medication use, and reproductive and medical history. Plasma was obtained for HIV-1 RNA quantification. A pelvic examination was performed with collection of vaginal swabs for diagnosis of vaginal infections and *Lactobacillus* culture. Vaginal fluid specimens were not collected if the participant was menstruating. Bacterial vaginosis was diagnosed from vaginal Gram stain using Nugent’s criteria [7]. *Trichomonas vaginalis* was detected by culture using the InPouch system (Biomed Diagnostics, White City, Oregon). The presence and quantity of *Lactobacillus* was assessed by vaginal culture on Columbia 5% sheep blood and Rogosa agar. Isolates from blood agar were grown in 5-10% CO_2_. Isolates from Rogosa agar were incubated anaerobically. All colonies with morphology suggestive of *Lactobacillus* on blood agar as well as any colonies growing on Rogosa were isolated and identified on the basis of colony morphology and Gram stain [8,9]. These isolates were subcultured on tetramethylbenzidine (TMB) agar containing horseradish peroxidase in order to assess hydrogen peroxide (H_2_O_2_) production [10,11].

Cervicovaginal lavage (CVL) was collected by washing the ectocervix and vaginal walls with 7 mL of 10 mM lithium chloride solution, which was then collected from the vaginal pool, spun at 800 g for 5 minutes to separate the epithelial cells and stored at −80°C. Plasma and CVL HIV-1 RNA were quantified by an independently validated real-time PCR assay described previously [12], with a lower limit of detection of 30 copies/mL.

The frozen cell pellet from CVL was thawed and underwent DNA extraction with the MO BIO Bacteremia Isolation Kit (MO BIO Laboratories, Inc., Carlsbad, CA). Extracted DNA was tested by quantitative PCR using primers targeting the human 18S rRNA gene to validate that successful DNA extraction occurred and an internal amplification control PCR using exogenous DNA from a jellyfish gene was used to test for presence of PCR inhibitors [13]. Samples were then subjected to taxon-directed 16S rRNA gene qPCR assays for the detection and quantification of *L. crispatus* and *L. jensenii*. Each assay had been validated previously and proven to be sensitive (to a level of 1–10 DNA copies/reaction) and specific (does not detect other bacteria at a concentration of 10^6^ copies/reaction) [14,15]. The assays used a TaqMan format and were run on an ABI 7500 Thermocycler (Applied Biosystems, Foster City, CA) or Eppendorf Mastercycler ep Realplex thermal cycler (Eppendorf, Westbury, NY). Negative assays were assigned a value at the lower limit of detection for that assay.

Based on the findings from our analysis of concordance, we conducted an exploratory analysis using broad range 16S rRNA gene PCR on a subset of culture isolates derived from samples with high quantities of H_2_O_2_-producing *Lactobacillus* by culture (≥ 10^6^ colony forming units [CFUs]), but with negative results for both *L. crispatus* and *L. jensenii* by qPCR in CVL. *Lactobacillus* isolates were retrieved from −80°C storage and streaked to Rogosa agar and Columbia agar with 5% sheep blood. A repeat streak to fresh agar was performed in order to obtain new isolates for broad range PCR testing. Genomic DNA was extracted from single bacterial colonies, single colonies converted to a lawn of bacteria (patches), or streaks of colonies on plates using the BiOstic Bacteremia DNA Isolation kit (MO BIO Laboratories, Inc., Carlsbad, CA). PCR was performed to amplify a portion of the bacterial 16S rRNA gene using combinations of the following broad range bacterial primers: 27 F bact (5'-AGAGTTTGATCMTGGCTCAG-3'), 338 F (5'-ACTCCTRCGGGAGGCAGCAG-3'), 806R (5'-GGACTACCAGGGTATCTAAT-3') and 1407R (5'-GACGGGCGGTGWGTRCA-3'). A range of 10ul neat DNA to 1ul 1:10 diluted DNA was added to 50ul reaction. Thermal cycling consisted of pre-melting at 95°C for 2 minutes, followed by 25 cycles of melting at 95°C for 30 seconds, annealing at 55°C or 60°C for 30 seconds and extension at 72°C for 2 minutes, with a final extension at 72°C for 10 minutes. PCR products were cleaned using the 0.5-mL 100 K Amicon Ultra Centrifugal Filters (Millipore, Billerica, MA). The same PCR primers were used in the sequencing reactions using ABI Prism 3730xI DNA Analyzer and BigDye chemistry. Sequencher software (GeneCodes, Ann Arbor, MI) was used to assemble sequence data. Sequences of the isolates (~800 base pairs in length) were compared to known bacterial sequences in Genbank by BLAST algorithm. Identities of the isolates to known species was defined by having >98% sequence similarity.

We used descriptive statistics for planned and exploratory analyses and calculated a kappa statistic to assess concordance between culture and qPCR using Stata version 11.0 (StataCorp, College Station, TX).

## Results

Culture and qPCR results were available for 376 visits from 57 women. The median number of visits per woman was 6 (interquartile range (IQR): 3–6; range: 1–15). The median age of participants at enrollment was 38 (IQR: 35–42) and 33 (58%) reported African-American race. The median CD4 count and plasma viral load was 417 cells/mm^3^ (IQR: 254–639) and 370 copies/mL (IQR: <30 – 15, 850), respectively. Over half of women were on antiretroviral therapy at enrollment and BV by Gram stain was present among 24 (42%) women.

Lactobacilli were detected by culture at 308 (82%) visits, of which 233 of 308 (76%) produced H_2_O_2_ . The median quantity of H_2_O_2_+ *Lactobacillus* was 1.0E + 10^7^ CFUs (IQR: 4.0E + 10^5^, 2.0E + 10^8^) among visits where *Lactobacillus* was detected by culture. *L. crispatus* was detected by qPCR at 173 (46%) visits. Among visits with detectable *L. crispatus*, the median quantity was 5.6E + 10^6^ gene copies per sample (IQR: 7.0E + 10^4^, 4.1E + 10^7^). *L. jensenii* was detected slightly less often at 130 (35%) of visits. Among visits with detectable *L. jensenii*, the median quantity was 10.7E + 10^5^ gene copies per sample (IQR: 2.2E + 10^5^, 7.1 + 10^6^). *L. crispatus* alone was detected at 85 (23%) visits and *L. jensenii* alone was detected at 42 (11%) visits. Both bacteria were present at 88 (23%) visits and either *L. crispatus* and/or *L. jensenii* were present at 215 (57%) visits. Concordance between detection of *L. crispatus* and/or *L. jensenii* by qPCR and H_2_O_2_-production by culture was 75% (kappa = 0.45), which equates to a modest level of agreement (Table 1).

**Table 1:** *** Lactobacillus *species detected by qPCR compared to H**_**2**_**O**_**2**_+* Lactobacillus *detected by culture*

**Species Detected by PCR**		**H**_**2**_**O**_**2**_**+*Lactobacillus*Detected by Culture**	
		**Detected n = 233**	**Not detected n = 143**
		N (%)	N (%)
*L. crispatus*	Detected	149 (64)	24 (17)
	Not detected	84 (36)	119 (83)
*L. jensenii*	Detected	107 (46)	23 (16)
	Not detected	126 (54)	120 (84)
*L. crispatus* and/or *L. jensenii*	Detected	174 (75)	41 (29)
	Not detected	59 (25)	102 (71)

*N = 376 samples.

Given the moderate level of agreement between culture and qPCR, we conducted an exploratory analysis using broad range PCR on a subset of culture samples that had high quantities of H_2_O_2_-producing *Lactobacillus* by culture, but were negative for both *L. crispatus* and *L. jensenii* in CVL by qPCR. Twenty-five samples from 13 different women met our inclusion criteria for the exploratory analysis (8 women had one sample, 4 women had 2 samples and 1 woman had 9 samples). We selected 16 samples to undergo additional broad range PCR testing: one sample from each participant with ≤2 eligible samples (n = 12) and 4 from the participant with 9 eligible samples. For participants with 2 eligible samples, the sample with the highest quantity of *Lactobacillus* by culture was selected. If the quantity of *Lactobacillus* by culture was the same, the first visit in the series was selected. For the participant with 9 eligible samples, the 4 with the highest quantities of *Lactobacillus* by culture were selected. The prevalence of *Lactobacillus* species detected by broad range PCR is presented in Table 2. *L. gasseri* was the dominant species and was present among 81% of samples, including all isolates from the participant who contributed 4 samples.

**Table 2:** Prevalence of* Lactobacillus *species detected using broad range PCR among a subset of culture samples*

**Species**	**Number of samples with species detected**^**1**^**n = 16**
*L. gasseri*	13 (81)
*L. crispatus*	2 (13)
*L. iners*^*2*^	2 (13)
*L. johnsonii*	2 (13)
*L. coleohominis*	1 (6)
*L. oris*	1 (6)
*L. reuteri*	1 (6)
*L. rhamnosus*	1 (6)

*Samples from women with high quantities of H_2_O_2_-producing *Lactobacillus* by culture (≥ 10^6^ CFUs), but were negative for both *L. crispatus* and *L. jensenii* in CVL by qPCR.

^1^Percent total >100% since several isolates contained more than one species.

^2^H_2_O_2_-functionality was reassessed for the 2 isolates that were identified as *L. iners*. No H_2_O_2_-production was observed.

## Discussion

Among HIV-1 seropositive women, 19% of samples with detectable *L. crispatus* or *L. jensenii* by qPCR were negative for H_2_O_2_-producing *Lactobacillus* by culture. It has been reported that 95% of *L. crispatus* isolates and 94% of *L. jensenii* isolates cultured from vaginal fluid were observed to be H_2_O_2_-producers [5]. The discordance observed in our initial analysis could be due to more frequent detection of H_2_O_2_-nonproducing strains of these species by qPCR, or the *L. crispatus* and *L. jensenii* strains that grow well in the laboratory may over-represent those that produce H_2_O_2_ compared to the original pool of bacteria. Conversely, a quarter of samples that were positive for H_2_O_2_-producing *Lactobacillus* by culture had undetectable quantities of *L. crispatus* and *L. jensenii* by qPCR. This finding may be due to the presence of other less prevalent *Lactobacillus* species such as *L. gasseri,* which is commonly an H_2_O_2_-producer [6]. This discordance prompted an exploratory analysis, which showed that *L. gasseri* was indeed the predominant species detected among women who had high quantities of H_2_O_2_-producing *Lactobacillus* by culture (≥ 10^6^ CFUs), but were negative for both *L. crispatus* and *L. jensenii* in CVL by qPCR.

*L. gasseri* has been associated with the presence of abnormal vaginal microbiota [8], which may explain its detection in the absence of *L. crispatus* and *L. jensenii,* two species that are associated with a normal vaginal microbiota [4]. Though the proportion of *L. gasseri* isolates that are H_2_O_2_-producers is reported to be high (71-82%) [5,6], the prevalence of *L. gasseri* in the vagina is relatively low compared to *L. crispatus* and *L. jensenii*[5,16]. It is possible that *L. gasseri* is more prevalent in this population of HIV-1 infected women; however, since species-specific assays for *L. gasseri* were not performed and broad range PCR was conducted on only a subset of samples we do not know the overall prevalence of *L. gasseri*.

Our exploratory analysis also revealed a number of other *Lactobacillus* species besides *L. crispatus, L. jensenii,* and *L. gasseri* that appear capable of H_2_O_2_ production (Table 2). Among HIV-1 seropositive women, detection of H_2_O_2_-producing lactobacilli in the vagina has been associated with a reduced likelihood of genital HIV-1 shedding [2,3]. In addition, several epidemiologic studies conducted among HIV-seronegative women have reported an association between the presence of H_2_O_2_-producing *Lactobacillus* and a decreased risk of genital tract infections, including HIV-1 [17-20], leading to the hypothesis that H_2_O_2_ may inhibit genital tract pathogens. However, recent evidence indicates that *in vivo* concentrations of H_2_O_2_ produced from *Lactobacillus* may not be sufficient to provide protection against genital tract infections [21,22]. Given the evidence from epidemiological studies, it is possible that H_2_O_2_ production may serve as a marker for other phenotypes that play a role in the suppression of HIV-1 genital shedding and protection against genital tract infections. Additional studies are needed to improve our understanding of the biological mechanisms that contribute to reductions in genital HIV shedding and protection against genital tract infections.

Until recently, our understanding of the bacterial species associated with BV was limited to those species that could be detected by culture. Molecular techniques that detect bacterial genes such as the 16S ribosomal RNA (rRNA) gene have been used to characterize the vaginal microbiota and have revealed several new species [4]. Broad range and species-specific quantitative PCR assays allow for detection of both cultivation-resistant and cultivatable bacterial species, thus providing a more comprehensive characterization of the vaginal microbiota. However, cultivation approaches continue to play an important role in the characterization of the vaginal microbiota, as they provide important phenotypic information. Compared to molecular methods, culture is inexpensive and requires less technical skill, time, and infrastructure to generate results. Information generated from the use of culture and molecular methods performed in combination will likely continue to generate useful information about how the vaginal microbiota impacts human health.

This analysis should be interpreted in the context of several limitations. Our analysis population consisted of HIV-1 seropositive women; therefore, our findings may not be generalizable to HIV-1 uninfected women. Since species level identification of *Lactobacillus* culture isolates was not performed, we can’t directly compare the species level concordance by culture and qPCR of CVL. We used species-specific qPCR assays that are highly sensitive and can detect species even at very low levels. However, we did not develop or apply assays for every known *Lactobacilus* species. Note that we did not attempt to correlate culture results with detection of *L. iners* since this bacterium is typically not an H_2_O_2_ producer [5,16,23].

## Conclusions

The two most prevalent H_2_O_2_-producing *Lactobacillus* species that colonize the vagina, *L. crispatus* and/or *L. jensenii*, were detected among three-quarters of specimens that had detectable H_2_O_2_-producing *Lactobacillus* by culture. However, one-quarter of specimens with detectable H_2_O_2_-producing *Lactobacillus* by culture were negative for *L. crisaptus* and *L. jensenii*. This discordance lends support to the use of both culture and molecular methods to characterize the vaginal microbiota. Culture plays an important role in characterizing the vaginal microbiota by determining the metabolic capabilities of these bacteria, while the use of molecular methods enables the detection of cultivatable and cultivation resistant species. The use of these methodologies in combination holds great promise for improving our understanding of the vaginal microbiota and the molecular epidemiology of BV.

## Competing interests

The authors declare no commercial or other associations that might pose a conflict of interest.

## Authors’ contributions

JEB, CM, and JH conceived of the study and participated in its design. JB performed the statistical analysis and drafted the initial manuscript. KA performed the culture assays. CL and TF performed the species-specific qPCR and broad range bacterial PCR assays in the lab of DNF who oversaw the performance of the qPCR assays and participated in the design of the study. SEC, AL and RC participated in the design of the original cohort study and participated in the design of the present study. All authors read and approved the final manuscript.

## This abstract was presented at the Annual Scientific Meeting and Symposium of the Infectious Diseases Society for Obstetrics and Gynecology, in Santa Fe, NM; August 5–7, 2010

## Pre-publication history

The pre-publication history for this paper can be accessed here:

http://www.biomedcentral.com/1471-2334/12/188/prepub
